# Denosumab Treatment Does Not Halt Progression of Bone Lesions in Multicentric Carpotarsal Osteolysis Syndrome

**DOI:** 10.1002/jbm4.10729

**Published:** 2023-03-09

**Authors:** Melissa A. Lerman, Michael Francavilla, Lindsay Waqar‐Cowles, Michael A. Levine

**Affiliations:** ^1^ Division of Rheumatology The Children's Hospital of Philadelphia and Department of Pediatrics, University of Pennsylvania Perelman School of Medicine Philadelphia PA USA; ^2^ Department of Radiology Whiddon College of Medicine, University of South Alabama Mobile AL USA; ^3^ Division of Endocrinology and Diabetes and Center for Bone Health The Children's Hospital of Philadelphia and Department of Pediatrics, University of Pennsylvania Perelman School of Medicine Philadelphia PA USA

**Keywords:** DENOSUMAB, MafB, MULTICENTRIC CARPOTARSAL OSTEOLYSIS SYNDROME, RANKL‐INHIBITOR

## Abstract

Here we report the use of denosumab, a monoclonal antibody against receptor activator of nuclear factor κB ligand (RANKL), as monotherapy for multicentric carpotarsal osteolysis syndrome (MCTO) in an 11.5‐year‐old male with a heterozygous missense mutation in *MAFB* (c.206C>T; p.Ser69Leu). We treated the subject with 0.5 mg/kg denosumab every 60–90 days for 47 months and monitored bone and mineral metabolism, kidney function, joint range of motion (ROM), and bone and joint morphology. Serum markers of bone turnover reduced rapidly, bone density increased, and renal function remained normal. Nevertheless, MCTO‐related osteolysis and joint immobility progressed during denosumab treatment. Symptomatic hypercalcemia and protracted hypercalciuria occurred during weaning and after discontinuation of denosumab and required treatment with zoledronate. When expressed in vitro*,* the c.206C>T; p.Ser69Leu variant had increased protein stability and produced greater transactivation of a luciferase reporter under the control of the *PTH* gene promoter than did wild‐type MafB. Based on our experience and that of others, denosumab does not appear to be efficacious for MCTO and carries a high risk of rebound hypercalcemia and/or hypercalciuria after drug discontinuation. © 2023 The Authors. *JBMR Plus* published by Wiley Periodicals LLC on behalf of American Society for Bone and Mineral Research.

## Graphical abstract

In Multicentric Carpotarsal Osteolysis syndrome (MCTO) with mutated MafB, treatment with denosumab improved bone mineral density, but it did not limit progression, as seen by increase in bone damage (erosions and joint space narrowing) and progressive limitation of joint range of motion.

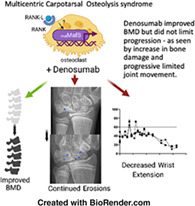

## Introduction

Multicentric carpotarsal osteolysis syndrome (MCTO; OMIM 166300) is an ultra‐rare disorder that usually presents in early childhood as an arthropathy that affects the wrists and ankles.^(^ ^1^ ^)^ MCTO is principally a localized osteolytic syndrome of the carpal and tarsal bones, but it may also involve other parts of the skeleton (eg, long bones, cervical spine, and craniofacial bones)^(^ ^2^, ^3^ ^)^ and affect extraskeletal tissues causing corneal clouding^(^ ^4^ ^)^ and focal segmental glomerulosclerosis (MCTO nephropathy).^(^ ^5^, ^6^, ^7^ ^)^ Previous work has identified heterozygous missense mutations in the V‐maf musculoaponeurotic fibrosarcoma oncogene homolog B (avian) (*MAFB*) gene, which encodes the transcription factor MafB, as the basis for MCTO.^(^ ^8^, ^9^, ^10^, ^11^, ^12^, ^13^, ^14^, ^15^ ^)^ Although MafB is widely expressed, the focal nature of the bone disease and the variable expressivity of the phenotype of MCTO even between affected individuals in the same family suggest that additional factors may modulate the MCTO phenotype (reviewed in^(^ ^1^ ^)^).

**Fig. 1:**
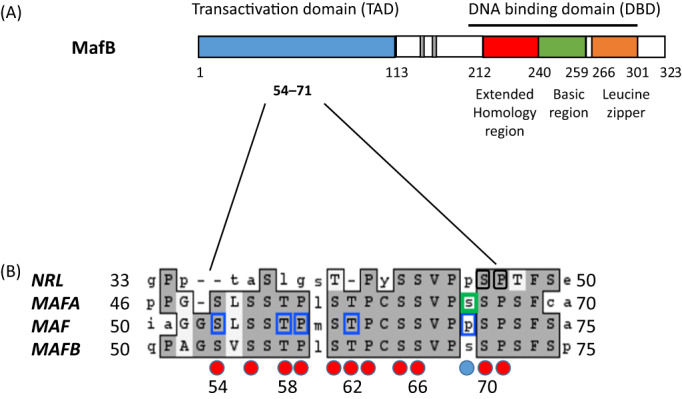
MAFB structure and associated MCTO mutations. (*A*) The upper panel shows the domain structure of human MafB protein, which comprises two main domains, an N‐terminal TAD and a C‐terminal DBD. The latter consists of an extended homology region (red), basic region (green), and leucine zipper (orange). The *MAFB* mutations reported to date in patients with MCTO have been exclusively clustered within the phosphorylation region (codons 54–71) in the TAD. Two regions representing histidine repeats (130–138 and 161–167) are shown in gray. (*B*) Amino acid alignments of the GSK3 motif among MAF orthologues. The priming Ser70 and four tandemly arranged phosphorylatable serine/threonine residues (Ser66, Thr62, Thr58, and Ser54) within the GSK3 recognition region at the N‐terminal TAD of MafB are noted. Amino acids that are replaced in MCTO patients are above red circles, with the affected codon in the patient described in this report above a blue circle. Mutations in homologous residues of the TAD of closely related genes encoding the large MAF proteins NRL (black boxes), MafA (green boxes), and Maf (blue boxes) are also shown that cause autosomal dominant retinitis pigmentosa, familial insulinomatosis, and Aymé‐Gripp syndrome, respectively. DBD = DNA‐binding domain; TAD = transactivation domain.

MafB is a member of the Maf family of basic leucine zipper (b‐Zip) transcription factors. The large Maf proteins (MafA, MafB, c‐Maf or v‐Maf, and NRL) have a b‐Zip structure, a motif for DNA binding and protein dimerization, and a transactivation domain (Fig. 1*A*).^(^ ^16^ ^)^ In MCTO, mutations lead to replacement of amino acids within a small region (residues 54–71) of the MafB transactivation domain that is highly conserved in all large MAF proteins (Fig. 1).^(^ ^10^, ^11^, ^14^, ^15^ ^)^ The serine and threonine residues in this region (Fig. 1*B*) are phosphorylated through a hierarchal process that is highly conserved among all the large Maf proteins.^(^ ^1^, ^17^, ^18^, ^19^, ^20^ ^)^ Phosphorylation of Maf proteins leads to increased transactivation activity,^(^ ^18^ ^)^ but at the same time, phosphorylation decreases protein stability via activation of ubiquitination^(^ ^21^ ^)^ and proteasomal degradation.^(^ ^17^, ^18^, ^22^, ^23^, ^24^ ^)^ MCTO has with similar features whether the actual mutations occur at phosphorylated or nonphosphorylated residues.^(^ ^1^ ^)^

In the hematopoietic system, *MAFB* is specifically expressed in the myeloid lineage that includes monocytes, macrophages, and osteoclasts. Early studies showed that MafB inhibited osteoclastogenesis induced by receptor activator of nuclear factor κB ligand (RANKL; the ligand for the RANK protein that is required for osteoclast development and function) at the transcriptional level by binding to transcription factors FOS and MITF.^(^ ^25^ ^)^ By contrast, other data supports a role for MafB in enhancing osteoclastic bone resorption, because bone mass was increased in mice in which *Mafb* was disrupted.^(^ ^1^, ^26^ ^)^

Because there are so few patients with MCTO, there are no controlled trials on therapeutic intervention. Based on the hypothesis that excessive osteoclastic activity is the basis for the bone lesions in MCTO, investigators have treated occasional patients with anti‐resorptive agents. Bisphosphonates have been used but evidence of a beneficial response is lacking (^(^ ^8^, ^27^, ^28^, ^29^ ^)^ and personal communication, Michael Whyte, MD, Division of Bone and Mineral Diseases, Department of Internal Medicine, Washington University School of Medicine, St Louis, MO, USA). The recent publication of two case reports^(^ ^9^, ^30^ ^)^ that described the partial skeletal responses of young MCTO patients to denosumab (Amgen, Thousand Oaks, CA, USA), a human monoclonal antibody that binds to and inactivates RANKL, provided the rationale for us to determine whether denosumab might be of benefit to an adolescent boy with MCTO under our care.

## Case Report

**Fig. 2:**
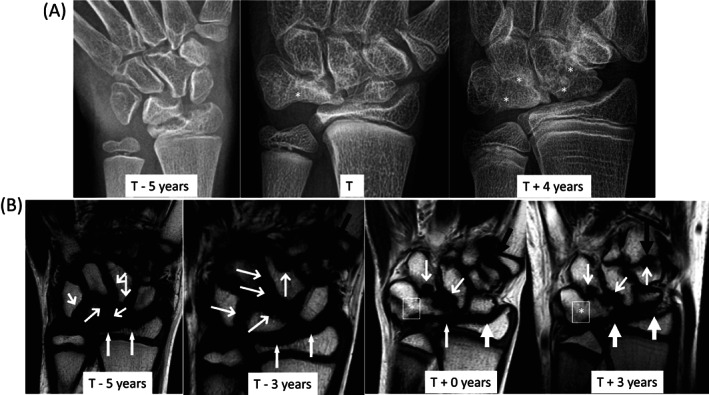
Images of left wrist. (*A*). Frontal radiographs of left wrist demonstrate multiple erosions in the distal radius, carpus, and proximal metacarpals with progressive carpal fusion (*) and loss of joint space. In posttreatment, overgrown distal radius and ulna epiphyses and multiple growth recovery lines are visible. (*B*) T1 signal‐weighted MR images of left wrist demonstrate to advantage numerous erosions in the radius (thick black arrows), ulna, carpals (thin black arrows), and metacarpal bases (white arrow). By 2017, the lunate and triquetrum have fused. By 2020, very little carpal joint space remains. MR = magnetic resonance.

The patient was referred to rheumatology at age 7.5 years for evaluation of left wrist pain that was noticed after a fall on the playground. He was adopted and no relevant family history was available. Physical examination revealed tall stature at 141 cm (*Z* = 2.71) with normal weight of 26.7 kg (Z = 0.52). He had swelling and tenderness of the left wrist, and radiography showed degenerative changes of the carpal bones, indicative of longstanding disease. All other joints were normal. Additional findings included micrognathia, hypertelorism with triangular face, and keloids from previous biopsies. A diagnosis of juvenile idiopathic arthritis (JIA) was made, and he was treated by intraarticular injection of corticosteroids. Because he showed no improvement to corticosteroid injections, he was subsequently treated with etanercept (Amgen, Thousand Oaks, CA, USA), a tumor necrosis factor α (TNFα) inhibitor, and later with methotrexate and tocilizumab (Genentech, South San Francisco, CA, USA), an interleukin 6R (IL‐6R) inhibitor, all with no benefit. Over time his joint disease progressed. He had decreasing range of motion of his right wrist, right hand, numerous fingers and both elbows, with magnetic resonance imaging (MRI) showing progressive narrowing of joint spaces and destruction of joint articular surfaces (Fig. 2). At age 10 years he underwent clinical whole‐exome sequence analysis (GeneDx, Gaithersburg, MD, USA) with the identification of a known pathogenic missense mutation in *MAFB* (c.206C>T, p.S69L) that confirmed the diagnosis of MCTO. At this point systemic anti‐inflammatory agents were discontinued. Comprehensive evaluation for MCTO revealed that, although he had many of the diffuse morphologic anatomic features of MCTO, he had normal renal function and did not have corneal clouding.

## Subject and Methods

### Treatment protocol

**Fig. 3:**
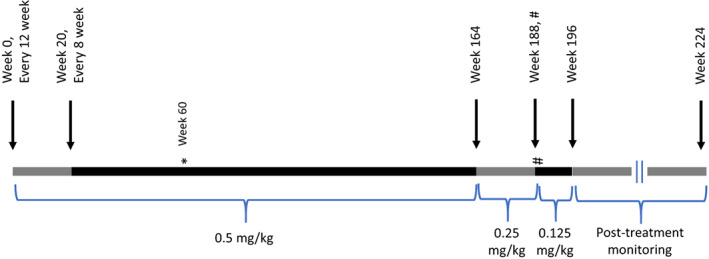
Denosumab dosing schedule. Horizontal bar shows time in weeks, colors change with change in: dosing interval or weight‐based dosing. Denosumab dosing (mg/kg) denoted below the bars. Arrows above the bars point to time of change (weeks at which change was made are included). *Indicates denosumab was 1 month late. #Indicates zoledronic acid treatment (0.125 mg/kg).

The protocol was approved by the Children's Hospital of Philadelphia (CHOP) Institutional Review Board and assent/consent was obtained from the subject/family. Denosumab was administered by subcutaneous injection at 0.5 mg/kg with an initial dosing interval of every 12 weeks that was changed to every 8 weeks after the first cycle (see Fig. 3). The patient received denosumab over a course of 47 months with a 3‐month suspension of therapy between months 22 and 25. Owing to concerns of hypercalcemia following denosumab discontinuation in children,^(^ ^31^, ^32^, ^33^, ^34^ ^)^ we tapered the dose of denosumab over the last 6 months of treatment (Fig. 3). Elemental calcium (500 mg twice daily) and calcitriol (1.5 μg twice daily) were administered orally with denosumab in the early months of the study, but due to persistently low serum levels of parathyroid hormone (PTH), the calcitriol was weaned and discontinued. The patient received 15 μg of cholecalciferol per day throughout the study.

### Clinical measurements

We monitored height with a stadiometer (Holtain, Crymych, UK) and weight with a digital scale (Scaletronix, White Plains, NY, USA). Joint swelling was assessed by physical examination and range of motion (ROM) was measured by goniometer.

### Biochemical testing

Serum and urine biochemistries were measured in the Clinical Laboratory at CHOP by standard techniques. Markers of bone turnover (procollagen type 1 N‐terminal propeptide [P1NP], bone‐specific alkaline phosphatase [BSAP], c‐telopeptide [CTX]) and cytokines were measured by enzyme‐linked immunosorbent assay (ELISA) techniques in commercial laboratories.

### Imaging

Skeletal lesions were evaluated by standard radiographs and MRI. Erosions in the wrist were scored using MRI based upon the 15‐site evaluation system proposed by Malattia and colleagues^(^ ^35^, ^36^ ^)^ and the simplified version of the Outcome Measures in Rheumatology Clinical Trials (OMERACT).^(^ ^37^ ^)^ Areal bone mineral density (BMD) was assessed by dual‐energy X‐ray absorptiometry (DXA) using a Hologic Horizon A scanner (Hologic, Bedford, MA, USA) and software version 13.5.3.1:3. The precision error for BMD and bone mineral content (BMC) at our institution is <1% for the spine. DXA *Z* scores were calculated^(^ ^38^ ^)^ and were adjusted for height *Z*‐score^(^ ^39^ ^)^ to yield BMD_haz_ *Z* scores.

### In vitro analyses of MafB expression and activity

We constructed a MafB‐responsive luciferase reporter by subcloning the 5.2‐kb BglII–BglII 5′‐regulatory fragment of the human *PTH* gene (kindly provided by Andrew Arnold, University of Connecticut)^(^ ^40^ ^)^ (GenBank AF346654), which contains four consensus Maf recognition sequence (MARE) sites,^(^ ^41^ ^)^ into the pGL3‐Luc reporter vector (Promega, Madison, WI, USA). We used a human MafB cDNA (NM_005461) in vector pCMV6‐XL5 (SC116756; Origene, Rockville, MD, USA) and created the p.S69L mutant by polymerase chain reaction (PCR) site‐directed mutagenesis as described.^(^ ^42^, ^43^ ^)^ The methods used to assess the effect(s) of the amino acid replacement on MafB stability and function are described in the Supporting Information.

### Statistical analyses

ROM is expressed as the mean of three consecutive measurements. Biochemical results are presented as the mean ± standard error of the mean (SE). Statistical significance for in vitro experiments was assessed by one‐way analysis of variance (ANOVA) with Student Neuman‐Keuls posttest per GraphPad InStat version 3.10 (GraphPad Software, San Diego, CA, USA). Data from all experiments were combined and analyzed. In all cases a *p* value of less than 0.05 was considered significant.

## Results

### Clinical outcomes

**Fig. 4:**
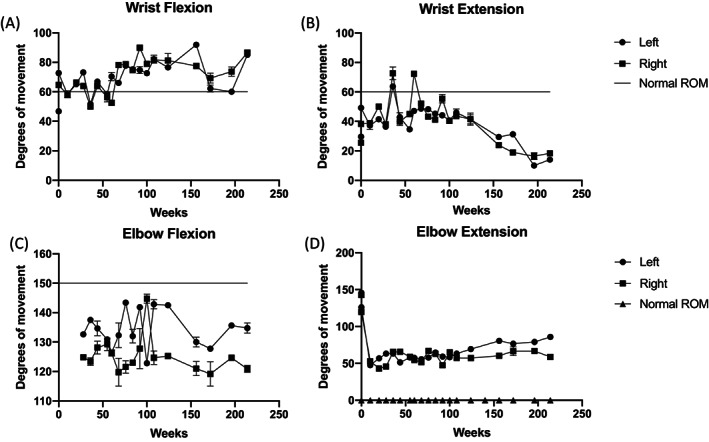
Wrist and elbow range of motion. Range of motion was measured with a digital goniometer and is shown over time (weeks). Mean of measurements taken in triplicate is shown; error bars (standard deviation). Wrist flexion (*A*), wrist extension (*B*), elbow flexion (*C*): • = left (line with circle), right (line with square), normal (straight line). elbow extension (*D*): • = left (line with circle), right (line with square), normal (line with triangle).

During treatment the subject showed a progressive reduction in the ROM of his wrists and elbows (Fig. 4*A‐D*, Fig. S1) that was most pronounced with extension. Prior to development of impaired ankle dorsiflexion (right > left [R > L]), the subject manifested gait abnormalities. Height and weight increased early after institution of denosumab and then more rapidly upon the onset of puberty. At completion of denosumab treatment, he had reached a height of 189.8 cm (*Z* = 2.18) and body mass index (BMI) of 23.5 kg/m^2^ (*Z* = 0.86) (Fig. S2). Renal function and blood pressure remained normal during the study.

### Skeletal lesions

**Fig. 5:**
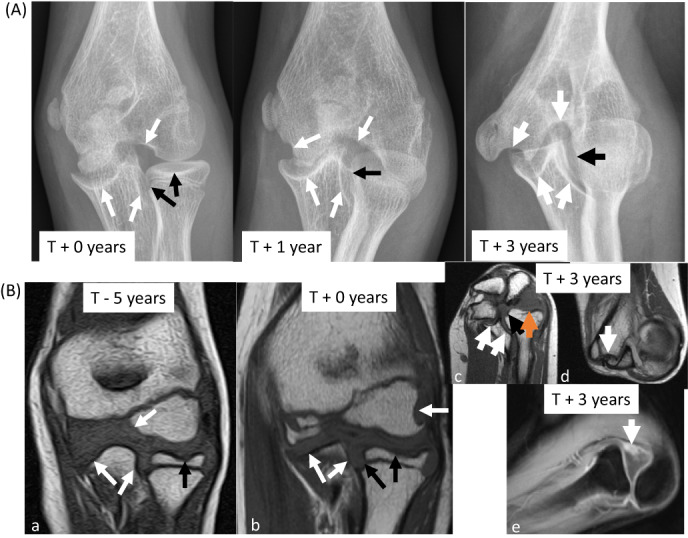
Images of left elbow. (*A*). Frontal radiographs of left elbow demonstrate multiple progressive erosions in the humerus and ulna (black arrows) as well as the radius (white arrows) with progressive deformity of the distal humerus and overgrowth of the subluxed radial head. (*B*) (a, b, c, d) T1 signal‐weighted MR images of left elbow demonstrate multiple progressive erosions in the humerus and ulna (blue arrows) as well as the radius (orange arrows) in the coronal plane (a, b). By 2020, the patient had a fixed flexion deformity that prevented acquisition in standard planes (c, d). (e) Post‐contrast T1 signal‐weighted image with fat saturation shows synovitis (arrow) and radiocapitellar subluxation. MR = magnetic resonance.

Pretreatment X‐rays revealed irregular orientation, margins, and erosive changes of the carpal bones with loss of normal carpal arcs as well as erosions of the distal radius and ulna. Over the course of the study, metacarpal base erosions worsened and then stabilized (Fig. 2*A*). By the second year, fusion had occurred between most of the carpal bones in both hands, the second metacarpal with the trapezoid in the right hand, and the lunate and triquetrum in the left wrist. During the study, extensive erosions progressed in the right elbow, although these largely resolved by study end (data not shown). The trochlea collapsed, the radial head became anteriorly subluxated and overgrown, and the lateral epicondyle become overgrown. Early on, joint space narrowing and erosions were present in the left elbow (Fig. 5*A‐B*). Over time there was increased collapse and fragmentation of the trochlea, epiphyseal overgrowth of the radial head and lateral humeral condyle and ultimately radial head subluxation.

The erosion score remained stable in the left wrist throughout the study (score: 18). Although a new scorable erosion developed at ~1.5 years, it then resolved. Synovial hyperenhancement and proliferation improved (Table 1, Fig. 2*B*). A slight joint effusion fluctuated independent of treatment. In the right wrist, the score initially increased (score: 22–23) and then decreased by study end (score: 21); this score did not reflect progressive ankylosis (Table S1). Although most erosions stabilized in the elbows, erosion of trochlea progressed bilaterally (Fig. 5, Tables S2 and S3). Joint inflammation, as manifested by a combination of effusion, synovial thickening, synovial enhancement, and bone marrow edema, improved in both elbows but did not fully resolve. Although the feet and ankles were minimally affected early on, new erosions developed (left medial malleolus and 3rd and 4th metacarpal bases; right medial cuneiform) with bone marrow edema in the left distal fibular epiphysis (Table S4).

**Table 1:** Description of MRI changes in left wrist, including OMERACT‐based scoring

Parameter	T0–2 w	T0 + 8 w	T0 + 36 w	T0 + 56 w	T0 + 76 w	T0 + 96 w	T0 + 128 w	T0 + 152 w
Total erosions	18	18; one ER improved	18	18; two small ER improved	18	19 distal ulna 0 → 1st MCP 1 → 3*	18 pisiform 1 → 0	18
Joint changes								
Synovial hyperenhancement	Mild	None	None	None	None	Poor quality	None	None
Synovial proliferation	Extensive				Mild		None	None
BME	New‐central scaphoid	None			Hamate	None	New scaphoid	
Joint effusion	Trace‐radioulnar	None	None	None	None	Trace		

*Note*: MRI with and without contrast. Erosive changes are scored according to the simplified version of the Outcome Measures in Rheumatology Clinical Trials MRI scoring system for erosions. Erosions scored at distal ulna, distal radius, triquetrum, pisiform, lunate, scaphoid, hamate, capitate, trapezoid, trapezium, 1st–5th MC bases. Erosions scored as: 0 = no erosion; 1 = 1%–25% of the bone eroded; 2 = 26%–50% of the bone eroded; 3 = 51%–75% of the bone eroded; and 4 = 76%–100% of the bone eroded. Absolute individual bone erosions scores not shown.

Abbreviations: BME = bone marrow edema; EN = enhancement; ER = erosion; MCP = metacarpal; MRI = magnetic resonance imaging; OMERACT = Outcome Measures in Rheumatology Clinical Trials; T_0_ = start of trial; T0−2 w = two weeks prior to treatment start date; T0  +  8 w = 8 weeks after treatment start; T0  +  36 w = 36 weeks after treatment start; T0  +  56 w = 56 weeks after treatment start; T0  + 76 w = 7 weeks after treatment start; T0  +  96 w = 96 weeks after treatment start; T0  +  12 w = 12 weeks after treatment start; T0  +  128 w = 128 weeks after treatment start; T0  +  152 w = 152 weeks after treatment start; w = week.

* Triquetrum is scored as lunate due to fusion. Cells are empty if no change from prior.

### Bone turnover

**Fig. 6:**
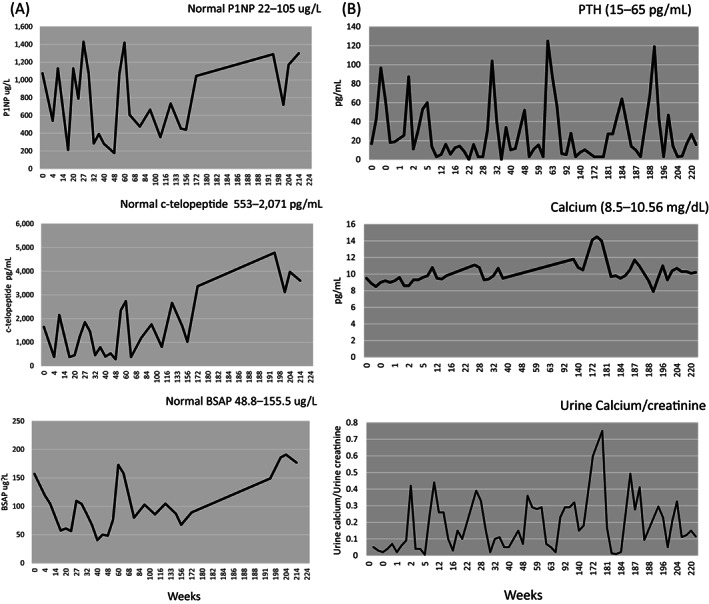
Biochemical markers. (*A*) Markers of bone formation: P1NP (reference range 22–105 ng/L); BSAP (reference range 48.4–155.5 until 1/16/19, then 27.8–210.9 ng/L); marker of bone resorption: CTX (reference range 553–2071 pg/mL until 1/16/19, then 485–2468). (*B*) PTH (reference range 12–65 pg/mL); serum calcium (reference range 8.5–10.6 mg/dL); urine calcium/urine creatinine (reference range less than 0.2 mg calcium/mg creatinine). BSAP = bone‐specific alkaline phosphatase; CTX = c‐telopeptide; P1NP = procollagen N‐terminal peptide; PTH = parathyroid hormone.

Prior to denosumab treatment serum levels of P1NP and BSAP were slightly greater than the upper limit of the age‐dependent reference ranges; serum levels of CTX were in the normal range for age (Fig. 6*A*). There was a marked decrease in all bone turnover markers after the first dose of denosumab; markers remained low at 8 weeks but rebounded to pretreatment levels when he was due for the second dose at week 12. Based on these observations we shortened the dosing interval to every 8 weeks. At the 20‐week mark, his P1NP had increased, but other bone turnover markers remained low. At the 28‐week mark, all bone turnover markers had increased. Anti‐denosumab antibodies were assessed and were negative (data not shown). With continued administration of denosumab every 8 weeks the bone turnover markers decreased and remained low based on age‐dependent reference ranges except for when a dose of study drug was delayed. All bone turnover markers increased as denosumab was weaned and then discontinued.

### Bone density

**Fig. 7:**
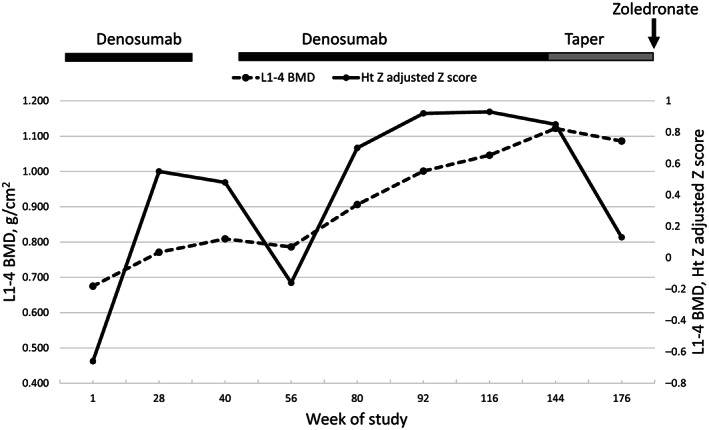
Bone densitometry. Changes in bone density of L_1_–L_4_ by DXA from baseline to month 48. Bone mineral density is shown on the left axis and the Ht *Z* adjusted *Z* score is shown on the right axis. Treatments are shown above the graphs. Ht = height.

Lumbar spine BMD was normal prior to treatment (BMD_haz_ *Z*‐score of −0.66) and increased after initiation of treatment (Fig. 7). Bone density declined after a brief period of discontinuation of denosumab and increased again when denosumab was restarted. Weaning of denosumab at the end of the treatment phase of the study led to a modest decrease in BMD.

### Systemic inflammation

Inflammatory markers (C‐reactive protein [CRP] and erythrocyte sedimentation rate [ESR]) and serum cytokines (IL‐2R, IL‐12, interferon γ [IFNγ], IL‐4, TNFα, IL‐2, IL‐5, IL‐10, IL‐13, IL‐1beta, IL‐6) were normal before and throughout the study.

### Biochemical studies of MafB

**Fig. 8:**
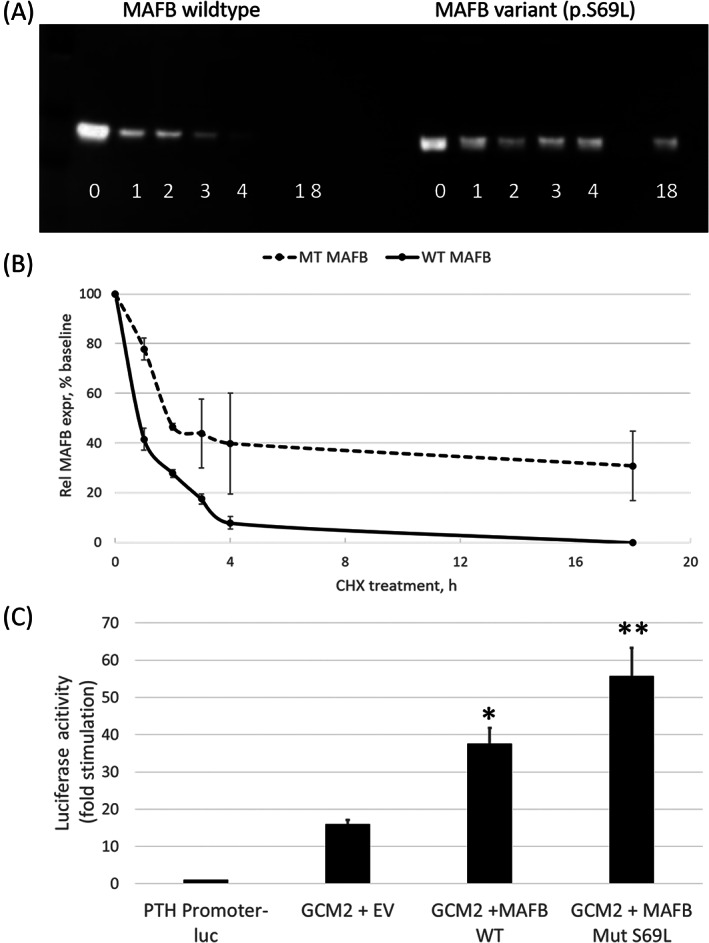
In vitro studies of MafB. Biochemical studies (*A*) MafB protein levels were determined by immunoblot analysis of lysates from HEK293T transfected with the indicated expression vectors after 1–18 hours of CHX treatment. The blot shown is representative of three different independent experiments. (*B*) Levels of MafB were calculated as the ratio of MafB protein to β‐actin after reprobing the membrane in *A* and scanning images as described in Methods. MafB half‐life is based on linear regression analysis of transformed *y* values. (*C*) MafB‐dependent MARE‐specific transcriptional activity in HEK293T cells transfected with the indicated MafB expression vectors or an EV plus GCM2. MARE‐specific luciferase activity is determined as the ratio of PTH promoter‐Luc reporter activity relative to that produced a *Renilla*‐luc reporter, expressed as fold stimulation over PTH‐luc only. *n* = 5, *p < 0.05 for WT versus EV and **p < 0.001 for S69L versus EV and WT. CHX = cycloheximide; EV = empty vector; WT = wild‐type.

Figure 8*A*,*B* shows that the half‐life for p.Ser69Leu is significantly greater than that for the wild‐type MafB protein (14.4 versus 1.1 hours, *p* < 0.01). In addition, HEK293T cells containing a luciferase reporter gene that is driven by the PTH gene promoter, which contains binding sites for GCM2 and MARE, showed greater luciferase activity after transfection with a plasmid expressing MafB p.Ser69Leu than cells that had been transfected with a plasmid encoding the wild‐type MafB protein in the presence (Fig. 8*C*) or absence (data not shown) of recombinant Glial cell missing 2 (GCM2).

### Safety analyses

The subject did not develop pathological fractures or bone pain during the study. He had a traumatic distal fibula fracture in the third year of the study, which healed well. Early on, the patient took calcium and calcitriol supplements for mild, asymptomatic hypocalcemia, but during later cycles calcitriol supplementation was discontinued because serum calcium levels remained normal.

The subject developed abdominal pain and nausea following the first decrease in denosumab dosing (from 0.5 to 0.25 mg/kg) at week 172 with elevated levels of urinary and serum calcium (Fig. 6*B*). This resolved with hydration, but symptomatic hypercalcemia recurred 6–7 weeks after the subsequent denosumab injection. Accordingly, he was maintained at 0.25 mg/kg for a third dose. Symptomatic hypercalcemia (abdominal pain) and hypercalciuria occurred prior to the next dose of denosumab (0.125 mg/kg) at week 188, and he was treated with zoledronic acid (0.5 mg/kg iv). He remained asymptomatic thereafter but continued to have mild hypercalcemia with suppressed PTH levels on the days of his second, and final 0.125 mg/kg denosumab injection. His mineral metabolism ultimately normalized and remained normal over the 9 months thereafter.

## Discussion

We found that denosumab treatment of our MCTO patient, with a p.Ser69Leu *MAFB* heterozygous mutation, over an approximately 4‐year period was associated with continued progression of MCTO despite increases in systemic bone mass. Given the lack of an untreated control we cannot be certain that denosumab did not at least reduce the pace of MCTO progression. Nevertheless, the lack of benefit in our patient is consistent with the limited results of denosumab treatment in two other MCTO patients who had been reported.^(^ ^9^, ^30^ ^)^ Regev and colleagues^(^ ^30^ ^)^ treated a single patient with MCTO, due to the same p.Ser69Leu variant as our patient, with denosumab 0.5–0.75 mg/kg every 4 months for 2 years. This patient had low BMD and elevated levels of serum RANKL (albeit measured while the patient was receiving denosumab), and denosumab treatment led to an increase in BMD, a decrease in joint pain, and stabilization—but no improvement—in osteolysis.^(^ ^30^ ^)^ Symptoms recurred when drug was discontinued. The patient was retreated for a short time 2 years later, and pain again improved. Zhuang and colleagues^(^ ^9^ ^)^ treated an 18‐year‐old female, with the p.Pro54Leu mutation, with a single 60‐mg dose of denosumab. A total of 9 months later repeat MRI showed improvement in inflammatory changes but no intervening imaging and clinical outcomes were provided. Important strengths of our study are that we closely monitored treatment of our patient with denosumab over a longer period of time than either prior study (3.9 years versus ~2 years) and used denosumab at greater doses and shorter intervals (every 2 months versus every 4 months) than in previous reports. Over this protracted treatment period denosumab did not reduce or prevent regional osteolysis or progressive loss of joint motion associated with radiographic bony fusion. Specifically, although some erosive bone lesions improved, new osteolytic lesions also developed during treatment and MRI erosion scores did not indicate improvement. Although some of the fluctuations in ROM may reflect measurement error, overall, there were trend toward worsening in wrist and elbow extension. This may be largely attributable to the fact that, even as erosions healed, bony overgrowth and fusion progressed, which further interfered with movement. Notably, as bone damage and crowding progressed more in the carpal bones than the distal radius and ulna, the damage may not have been reflected by measurement of wrist range of motion. Overall, the subject did not experience a clinically meaningful benefit.

Fewer than 30 cases of MCTO have been reported since 2012 and there do not appear to be any obvious genotype–phenotype distinctions among the 12 different *MAFB* missense mutations. Similar to the enhanced stability shown previously for MafB variants p.Ser54Leu, p.Pro63Leu, and p.Pro71Leu,^(^ ^44^ ^)^ our results show an increased half‐life of p.Ser69Leu relative to the wild‐type MafB protein. Moreover, the p.Ser69Leu variant led to increased transactivation of a MafB‐responsive reporter gene in HEK293T cells consistent with a gain‐of‐function. These results are similar to those reported for missense mutations affecting the homologous residues of the transactivation domain of closely related Maf, MafA, and NRL proteins (Fig. 1*B*) that occur in other disorders (ie, Aymé‐Gripp syndrome [AYGRPS],^(^ ^20^, ^45^, ^46^ ^)^ familial insulinomatosis,^(^ ^47^ ^)^ and autosomal dominant retinitis pigmentosa [RP27, MIM 613750],^(^ ^48^ ^)^ respectively). By contrast, heterozygous mutations in *MAFB* that cause haploinsufficiency or that generate dominant inhibitor proteins lead to the Duane Retraction syndrome‐1 (OMIM 126800) that, although not associated with skeletal lesions, does manifest focal segmental glomerulosclerosis.^(^ ^6^, ^7^, ^49^ ^)^

The mechanism of the skeletal lesions in MCTO remains uncertain. Preliminary studies of transgenic mice carrying a *Mafb* mutation that is homologous to the human MCTO mutation p.Pro59Leu show reduced bone density and increased osteoclastic activity compared to wild‐type mice, and bone marrow cells from these transgenic mice generate greater numbers of multi‐nucleated osteoclasts than cells from wild‐type mice after incubation with mCSF and RANKL (Takahashi, unpublished). Although we were unable to determine either local or serum levels of RANKL in our patient, serum levels of RANKL were elevated in the patient reported by Regev and colleagues^(^ ^30^ ^)^ (although they were measured while the patient was receiving denosumab). A direct effect of denosumab on RANKL‐dependent bone metabolism in MCTO is evidenced by the favorable BMD response to treatment in the patient we report here and in the patient treated by Regev and colleagues.^(^ ^30^ ^)^ What then could account for the inability of denosumab to affect the discrete osteolytic lesions of MCTO? One possibility is that we and others have administered doses/frequency of denosumab that are inadequate to inhibit RANKL‐mediated osteoclastic bone resorption in the highly aggressive localized lesions. For example, treatment protocols for giant cell tumor of the bone that achieve clinically significant responses utilize much greater doses and frequency of denosumab (ie, 120 mg administered subcutaneously every 28 days with multiple loading doses in month 1). Alternatively, it is possible that the pathogenic mechanism for the localized bone lesions in MCTO is unrelated to the one responsible for the systemic bone loss. A similar hypothesis has been proposed for Hajdu Cheney syndrome (OMIM 102500), in which craniofacial developmental abnormalities, acro‐osteolysis, and osteoporosis are due to gain‐of‐NOTCH2 function mutations,^(^ ^50^ ^)^ based on the observation that treatment of a single patient with denosumab was associated with progression of acro‐osteolysis but improvement of osteoporosis.^(^ ^51^ ^)^ It is therefore conceivable that the lack of response of the localized MCTO lesions to denosumab (or bisphosphonates) may reflect an underlying mechanism that is not dependent upon accelerated osteolysis. Lazarus and colleagues^(^ ^52^ ^)^ have proposed a model in which *MAFB* variants cause MCTO by disturbing the development of carpal, tarsal, and epiphyseal bones, and that altered bone formation rather than osteolysis accounts for the unique anatomic distribution of osseous abnormalities. Using wild‐type mice, these investigators showed that ossification of the carpal bones as well as the distal ulna and radius and second to fifth proximal metacarpals is characterized by a distinct variation of endochondral ossification that is highly dependent upon expression of MafB in areas of new bone formation.^(^ ^52^ ^)^ Consistent with this model, a recent preliminary report by others has shown that induced pluripotent stem cells that were derived from an MCTO patient with the Pro59Leu *MAFB* variant disrupted osteoblast and chondrocyte development.^(^ ^27^ ^)^ Moreover, compared to control cells, MCTO patient‐derived osteoblasts had decreased expression of OPN, OCN, OSX, and RUNX2 and patient‐derived chondrocytes had reduced expression of COL2 and aggrecan. Notably, if altered bone formation rather than bone destruction is the pathophysiology in MCTO, the customary use of the term erosion would be inappropriate. However, as the mechanism of altered bone morphology is not clear, and in the absence of other objective measures to describe bone lesions, we applied a scoring system for erosive lesions created for inflammatory arthritis.

During treatment, our subject tolerated denosumab without any untoward effects and demonstrated significant reductions in bone turnover markers. Not unexpectedly, bone turnover markers increased rapidly after discontinuation of denosumab, and our patient experienced symptomatic hypercalcemia. In adults, discontinuation of long‐term denosumab has been associated with consequent loss of BMD and the development of multiple vertebral compression fractures.^(^ ^53^ ^)^ In younger patients discontinuation of denosumab is associated with rebound hypercalcemia and hypercalciuria.^(^ ^31^, ^32^, ^33^, ^34^ ^)^ Our experience highlights this risk of prolonged use of denosumab in the pediatric population and further shows that rapid decreases in BMD can occur as well, raising the question of whether these young patients may also be at increased risk for the multiple vertebral fractures that occur in many older adults after discontinuation of denosumab.^(^ ^54^ ^)^

In conclusion, despite initial optimism that denosumab might halt the morbidity of MCTO, our experience adds to growing evidence that treatment with anti‐resorptive agents is unable to arrest the focal osteolytic disease. Continued work will be necessary to identify the relevant disease pathways that cause the localized skeletal lesions in MCTO in order to facilitate the development of effective targeted therapies.

## Author Contributions

**Melissa A. Lerman:** Conceptualization; data curation; formal analysis; funding acquisition; investigation; methodology; project administration; writing – original draft; writing – review and editing. **Michael Francavilla:** Formal analysis; writing – review and editing. **Lindsay Waqar‐Cowles:** Data curation; project administration; writing – review and editing. **Michael A Levine:** Conceptualization; formal analysis; writing – original draft; writing – review and editing.

## CONFLICT OF INTEREST

The authors have nothing to disclose.

### Peer Review

The peer review history for this article is available at https://publons.com/publon/10.1002/jbm4.10729.

## Supporting information


**Appendix S1.** Supporting information.
Supplemental Data.
Table S1.
Table S2.
Table S3.
Table S4.
Fig. S1.
Fig. S2.

## Data Availability

Raw data were generated at the Children's Hospital of Philadelphia. Derived data supporting the findings of this study are available from the corresponding author MAL on request.
