# Supplementary material for: Denosumab Treatment Does Not Halt Progression of Bone Lesions in Multicentric Carpotarsal Osteolysis Syndrome
Source: JBMR Plus. 2023 Mar 9;7(5):e10729. doi: 10.1002/jbm4.10729 (PMC10184019; doi:10.1002/jbm4.10729)
Supplement: Supplementary file 1 — Appendix S1. Supporting information. Supplemental Data. Table S1. Table S2. Table S3. Table S4. Fig. S1. Fig. S2. [file JBM4-7-e10729-s001.zip › jbm410729-sup-0001-supinfo/jbm410729-sup-0001-supinfo1.docx]

**Supplemental Data**

***Methods***

To determine the half-life of recombinant MafB proteins, we transiently expressed cDNAs in HEK293T cells that were grown in complete DMEM with cycloheximide (100 μg/mL) and collected lysates at the indicated times. Whole cell extracts were subjected to immunoblot analysis using a mouse anti-MaFB monoclonal antibody (Origene, Clone ID: OTI2A6], catalog number CF800291; used at 1:2000) against amino acids 32-320 of human MafB (NP_005452) and a mouse anti-β actin monoclonal antibody (AC-15, Sigma-Aldrich) to ensure equal loading. Antibody binding was detected by use of peroxidase-labeled secondary antibodies and was visualized using enhanced chemiluminescence following the manufacturer’s instructions (Pierce Chemical Co., Rockford, IL) and quantified by scanning as previously described (1).

To analyze transcriptional activity of wild type and mutant MafB, we transfected HEK293T cells in 24-well plates with the 100 ng of *PTH* promoter luciferase reporter, 100 ng of MafB plasmid, 100 ng of a wild type GCM2 plasmid (2), plus 10 ng/well of a Renilla luciferase reporter vector DNA driven by a minimally active thymidine kinase promoter (pRL-TK; Promega, Madison, WI). Luciferase activity was measured using Promega’s Dual-Luciferase Reporter Assay kit, and the ratio of firefly to Renilla luciferase activity was calculated as previously described (3,4). The data shown here represent the mean ± SEM for four separate experiments and are expressed as fold stimulation above the activity for the *PTH* promoter-luciferase vector alone.

***Supplemental Figures***

**Figure S1.** **Ankle Range of motion**. Range of motion was measured with a digital goniometer and is shown over time (weeks). Mean of measurements taken in triplicate is shown; error bars (standard deviation). Ankle plantarflexion (S1A), Ankle Dorsiflexion (S21B): • = Left (line with circle), Right (line with square), normal (straight line).

**Figure S2**. Growth Over Time. BMI (kg/m2) graphed against age in years (x) axis overlaid on Z scores from the CDC Boys 2-20 reference data. Arrows show: study drug initiation (week 0); first time dose was decreased to 0.25 mg/kg (week 164).

***Supplemental Tables:***

**Table S1.** **MRI changes of right wrist, both elbows, ankles**.

Legend: MRI with and without contrast of right wrist. Erosive changes are scored according to the simplified version of the Outcome Measures in Rheumatology Clinical Trials MRI scoring system for erosions. Erosions scored at distal ulna, distal radius, triquetrium, pisiform, lunate, scaphoid, hamate, capitate, trapezoid, trapezium, 1^st^🡪5^th^ MC bases. Erosions scored as: 0 = no erosion; 1 = 1-25% of the bone eroded; 2 = 26-50% of the bone eroded; 3 = 51-75% of the bone eroded; and 4 = 76-100% of the bone eroded. Absolute individual bone erosions scores not shown. T_0_=start of trial. w=week. MetaCP=metacarpal. BME=bone marrow edema. EN=enhancement. ER=erosion. *Limited by motion. ^†^ Erosions improved at lunate, scaphoid, hamate, capitate, trapezium, 1^st^,2^nd^, 3^rd^ metaCPs. Cells are empty if no change from prior.

**Table S2.** **MRI changes of right elbow.**

Legend: MRI with and without contrast of right elbow. T_0_=start of trial, w=week. BME=bone marrow edema. EN=enhancement. ER=erosion. JN= joint space narrowing. CL= cartilage loss. CT=cartilage thinning. OC= Ossification center. * All evaluations limited due to inability to fully extend, cartilage particularly limited. † Motion artifact. ** compared to 10/2016. †† Intermediate T1 rather than low T1, different from other BM edema – may be benign bone lesion development. Cells are empty if no change from prior.

**Table S3**. **MRI changes of left elbow**

Legend: MRI with and without contrast left elbow. T_0_=start of trial, w=week. BME=bone marrow edema. EN=enhancement. ER=erosion. JN= joint space narrowing. CL= cartilage loss. CT=cartilage thinning. OC= Ossification center. *Evaluation limited due to inability to fully extend, cartilage particularly limited. † Motion degraded in some sequences. **Compared to 10/2016. †† Failed development trochlear cartilage while other parts overgrown. Cells are empty if no change from prior.

**Table S4.** MRI changes of ankles

Legend. MRI with and without contrast of ankles. T_0_=start of trial, w=week. BME=bone marrow edema. EN=enhancement. ER=erosion. ML=malleolus. CL = calcaneous. MT=Metatarsal base. **Area never imaged before, so may not be new. Cells are empty if no change from prior.

**Supplemental References:**

1. Hanley PC, Kanwar HS, Martineau C, Levine MA 2020 Short Stature is Progressive in Patients with Heterozygous NPR2 Mutations. J Clin Endocrinol Metab **105**(10).

2. Maret A, Ding C, Kornfield SL, Levine MA 2008 Analysis of the GCM2 gene in isolated hypoparathyroidism: a molecular and biochemical study. J Clin Endocrinol Metab **93**(4)**:**1426-32.

3. Roizen JD, Li D, O'Lear L, Javaid MK, Shaw NJ, Ebeling PR, Nguyen HH, Rodda CP, Thummel KE, Thacher TD, Hakonarson H, Levine MA 2018 CYP3A4 mutation causes vitamin D-dependent rickets type 3. J Clin Invest **128**(5)**:**1913-1918.

4. Thacher TD, Fischer PR, Singh RJ, Roizen J, Levine MA 2015 CYP2R1 Mutations Impair Generation of 25-hydroxyvitamin D and Cause an Atypical Form of Vitamin D Deficiency. J Clin Endocrinol Metab **100**(7)**:**E1005-13.

**Appendix/Supplemental Tables**

Table S1: **Description of MR changes in right wrist, including OMERACT scoring**

| **Right Wrist** | **T_0_ -2 w** | **T_0_ +8 w** | **T_0_ +40 w** | **T_0_ + 96 w** | **T_0_ + 156 w** |
| --- | --- | --- | --- | --- | --- |
| Total Erosions | 22 | 23 | 23 | 21^†^  8 areas, improved | 21 |
| Joint changes |  |  | Fusion lunate and triquetrum | Worsening fusion carpal, and carpal- 2^nd^ MC | Progressive fusion carpal and carpal-MC |
| Synovial hyperenhancement | None |  |  | None * |  |
| Synovial proliferation | None |  |  |  |  |
| BM edema | Tiny focus distal radius |  |  | None * |  |
| Joint effusion | None |  |  | Trace |  |

Table S2: **Description of MR changes in right elbow**

| **Right Elbow*** | **T_0_** † ** | **T_0_ +8 w** | **T_0_ +40 w** | **T_0_ +96 w** | **T_0_ +152 w** |
| --- | --- | --- | --- | --- | --- |
| Erosion | Worsening ER central radial head, ulnar margin capitellum, and sublime tubercle.  Stable ER radial margin capitellum.  CL radial head. | Increased ER capitellum and sublime tubercle. |  | Significantly improved ER at margins of radial head/neck articulation |  |
| Deformity | Progression of CT and JN at trochlear ulnar articulation.  Progressive collapse trochlear OC. Mild volar subluxation radius (to capitellum) | Improved radial head subluxation. Mild persistent volar radial head subluxation.  Progressive collapse trochlear OC. | Improved radial head subluxation; mild persistent volar radial head subluxation. Trochlear OC no longer identifiable. | increased volar radial head subluxation |  |
| Synovial hyperenhancement | Diffuse moderate |  |  | Mild, improved. |  |
| Synovial proliferation | Diffuse moderate |  |  | Minimal | Mild, stable |
| BM edema | Present in trochlea and olecranon (stable) | Some stable, some improved | Increased intercondylar distal humerus and posterior capitellum | New lesion radial margin lateral condyle ††  Others decreased or resolved |  |
| Joint Effusion | Moderate (stable) |  |  | Small |  |

Table S3: **Description of MR changes** of left elbow

| **Left Elbow *** | **T_0_** † ****** | **T_0_ +8 w** | **T_0_ +24 w** | **T_0_ +40 w** | **T_0_ +52 w** | **T_0_+76 w** | **T_0_ +100 w** | **T_0_ +120 w** | **T_0_ +148 w** |
| --- | --- | --- | --- | --- | --- | --- | --- | --- | --- |
| Erosion |  | Stable ER radial head and capitellum |  |  |  |  |  | New ER- lateral humeral condyle |  |
| Deformity | Trochlear cartilage††. Increased deformity distal humerus. Remodeling proximal ulna/coronoid | Stable diffuse JN and CL | Stable JN and cartilage loss | Unable to identify trochlear OC  Stable JN and cartilage loss |  |  |  |  |  |
|  |  |  |  |  |  |  |  |  |  |
| Synovial hyper-enhancement | Extensive (increased) | Moderate (stable) | Moderate (stable) | Moderate (stable) | Less |  | None | Mild | Mild |
| Synovial proliferation | Extensive | Moderate (stable) | Moderate (stable) | Moderate (stable) |  |  | Improved | None | None |
| BM edema | minimal | Increased (capitellum and distal humerus) | Some areas markedly improved, others stable. | Worsened: posterior lateral condylar metaphysis;medial condyle/  trochlea. | Improved |  | Less intense | New- posterior lateral distal humerus. Olecranon improved. | Resolution of new BME from 2/2020 |
| Joint Effusion | Moderate (increased) | Moderate (Stable) | Moderate (Stable) | Smaller |  | Larger | Stable (small) | Stable (small) | Mildly smaller |

Table S4. **Description of MR changes** of ankles.

| **Left Ankle** | **T_0_ -2 w** | **T_0_ +8 w** | **T_0_ +40 w** | **T_0_ +100 w m** | **T_0_ +112 w** | **T_0_ +148 w** |
| --- | --- | --- | --- | --- | --- | --- |
| Erosion | None ankle | Stable small: medial cuneiform/2^nd^ MT |  | New ER medial ML with EN | Stable ER medial ML, less EN  New 3^rd^ and 4^th^ MT ** | 1st description ER 1^st^ MT epiphysis and metaphysis |
| Synovial hyperenhancement |  | None |  |  |  |  |
| Synovial proliferation |  | None |  |  |  |  |
| BM edema | Mild (lateral fibular epiphysis) | Resolved |  |  |  | New: lateral distal fibular epiphysis |
| Joint Effusion |  | None |  | None | Trace subtalar | None |

| **Right Ankle** | **T_0_ -2w** | **T_0_ +4 w** | **T_0_ + 40 w** | **T_0_ + 100 w** | **T_0_ +120 w** | **T_0_ +148 w** |
| --- | --- | --- | --- | --- | --- | --- |
| Erosion | ER Distal medial ML | ER: stable ML, less EN CL. Stable cuneiform and cuboid. New ER 2^nd,^ 3^rd^  MT | New mild EN at MT) | New medial signal change cuneiform anteriorly | Stable ER and EN ML/CL, cuneiform, 2^nd,^ 4^th^ 5^th^ MT.  New ER medial cuneiform with EN |  |
| Synovial hyperenhancement | Present | Improved | None |  |  |  |
| Synovial proliferation | Present | Improved | None |  |  |  |
| BM edema | None |  | None |  |  |  |
| Joint Effusion | None |  |  |  |  |  |
