# Supplementary figures and images for: Denosumab Treatment Does Not Halt Progression of Bone Lesions in Multicentric Carpotarsal Osteolysis Syndrome
Source: JBMR Plus. 2023 Mar 9;7(5):e10729. doi: 10.1002/jbm4.10729 (PMC10184019; doi:10.1002/jbm4.10729)

## Slide 1
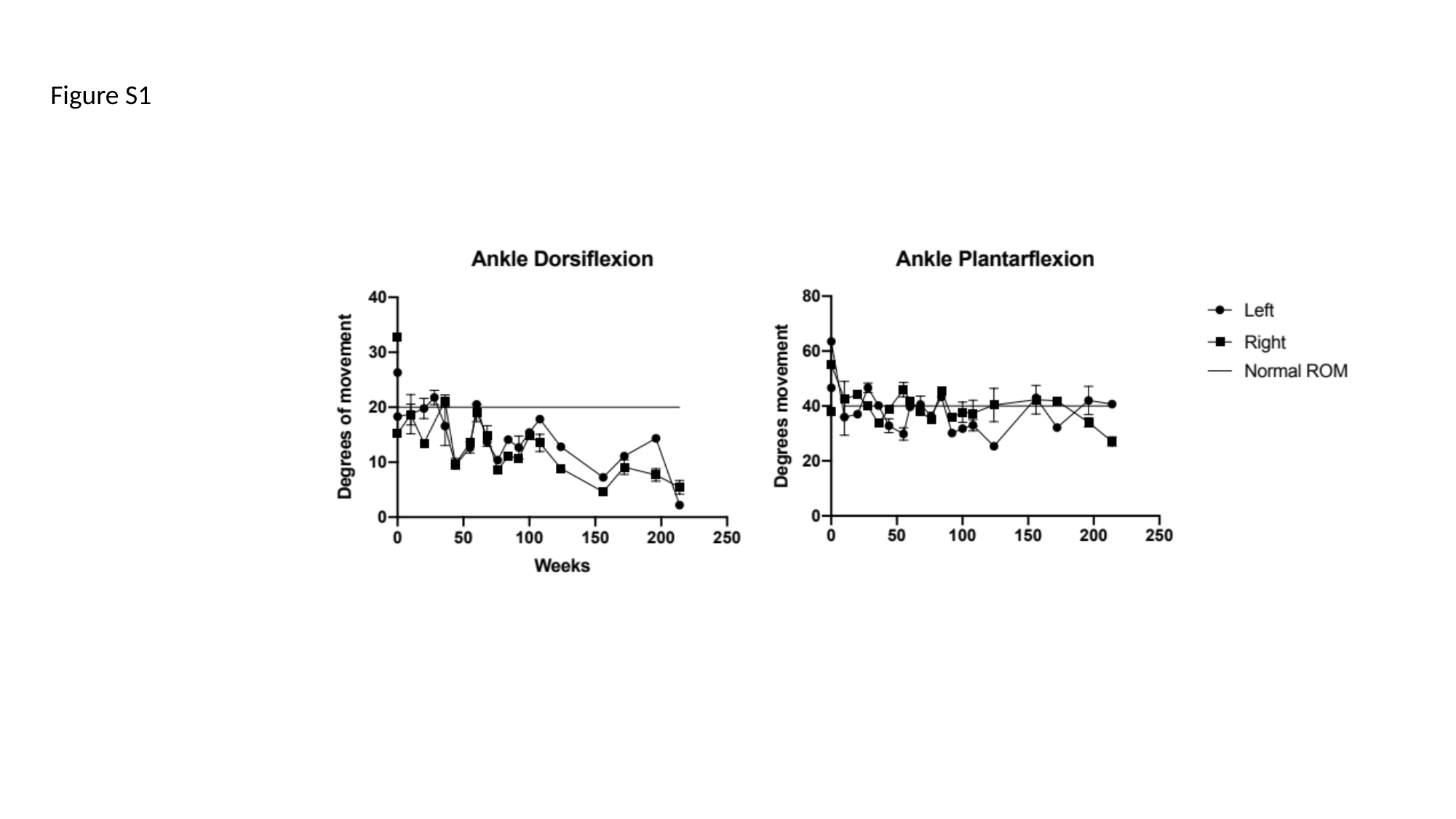

Figure S1

## Slide 2
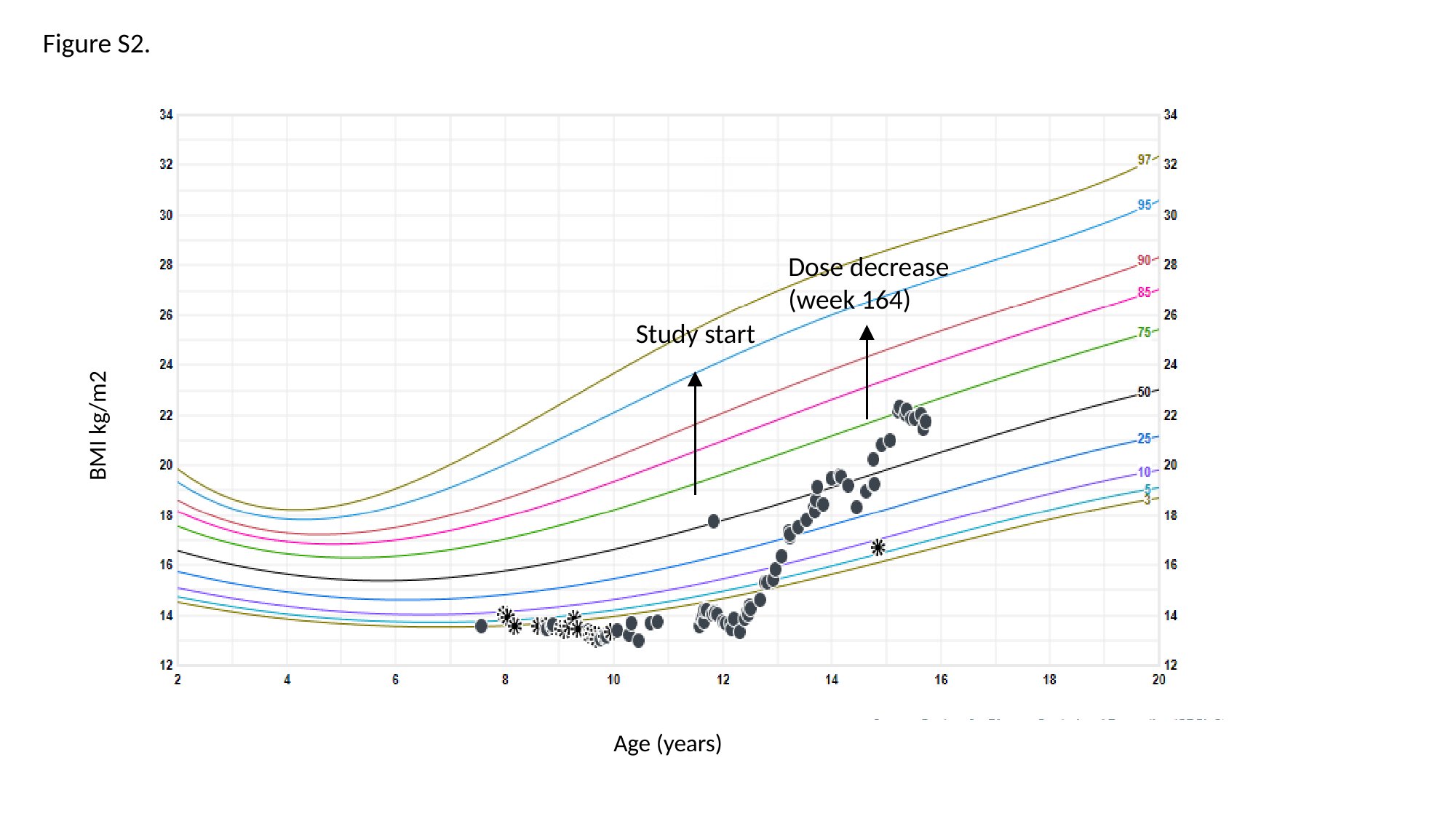

Figure S2.
Dose decrease
(week 164)
Study start
BMI kg/m2
Age (years)

Supplement: Supplementary file 1 — Appendix S1. Supporting information. Supplemental Data. Table S1. Table S2. Table S3. Table S4. Fig. S1. Fig. S2. [file JBM4-7-e10729-s001.zip › jbm410729-sup-0001-supinfo/jbm410729-sup-0002-supinfo2.pptx]
